# Astronauts Plasma-Derived Exosomes Induced Aberrant EZH2-Mediated H3K27me3 Epigenetic Regulation of the Vitamin D Receptor

**DOI:** 10.3389/fcvm.2022.855181

**Published:** 2022-06-16

**Authors:** Malik Bisserier, Agnieszka Brojakowska, Nathaniel Saffran, Amit Kumar Rai, Brooke Lee, Matthew Coleman, Aimy Sebastian, Angela Evans, Paul J. Mills, Sankar Addya, Arsen Arakelyan, Venkata Naga Srikanth Garikipati, Lahouaria Hadri, David A. Goukassian

**Affiliations:** ^1^Icahn School of Medicine at Mount Sinai, Cardiovascular Research Institute, New York, NY, United States; ^2^Department of Emergency Medicine, The Ohio State University Wexner Medical Center, Columbus, OH, United States; ^3^Lawrence Livermore National Laboratory, Livermore, CA, United States; ^4^Department of Radiation Oncology, University of California, Davis, Sacramento, CA, United States; ^5^Center of Excellence for Research and Training in Integrative Health, University of California, San Diego, La Jolla, CA, United States; ^6^Kimmel Cancer Center, Sidney Kimmel Medical College, Thomas Jefferson University, Philadelphia, PA, United States; ^7^Bioinformatics Group, Institute of Molecular Biology, National Academy of Sciences of the Republic of Armenia (NAS RA), Yerevan, Armenia; ^8^Department of Bioengineering, Bioinformatics, and Molecular Biology, Russian-Armenian University, Yerevan, Armenia; ^9^Dorothy M. Davis Heart Lung and Research Institute, The Ohio State University Wexner Medical Center, Columbus, OH, United States

**Keywords:** astronauts, spaceflight, EZH2, vitamin D receptor, small extracellular vesicles, epigenetic

## Abstract

There are unique stressors in the spaceflight environment. Exposure to such stressors may be associated with adverse effects on astronauts' health, including increased cancer and cardiovascular disease risks. Small extracellular vesicles (sEVs, i.e., exosomes) play a vital role in intercellular communication and regulate various biological processes contributing to their role in disease pathogenesis. To assess whether spaceflight alters sEVs transcriptome profile, sEVs were isolated from the blood plasma of 3 astronauts at two different time points: 10 days before launch (L-10) and 3 days after return (R+3) from the Shuttle mission. AC16 cells (human cardiomyocyte cell line) were treated with L-10 and R+3 astronauts-derived exosomes for 24 h. Total RNA was isolated and analyzed for gene expression profiling using Affymetrix microarrays. Enrichment analysis was performed using Enrichr. Transcription factor (TF) enrichment analysis using the ENCODE/ChEA Consensus TF database identified gene sets related to the polycomb repressive complex 2 (PRC2) and Vitamin D receptor (VDR) in AC16 cells treated with R+3 compared to cells treated with L-10 astronauts-derived exosomes. Further analysis of the histone modifications using datasets from the Roadmap Epigenomics Project confirmed enrichment in gene sets related to the H3K27me3 repressive mark. Interestingly, analysis of previously published H3K27me3–chromatin immunoprecipitation sequencing (ChIP-Seq) ENCODE datasets showed enrichment of H3K27me3 in the VDR promoter. Collectively, our results suggest that astronaut-derived sEVs may epigenetically repress the expression of the VDR in human adult cardiomyocytes by promoting the activation of the PRC2 complex and H3K27me3 levels.

## Introduction

With the rapid expansion of the commercial aerospace industry and future deep-space exploration to the Moon and Mars, space travel is now entering a new area. Thus, it is critical to understand the short- and long-term health risks associated with the space environment during low Earth orbit (LEO) space missions, such as those onboard the International Space Station (ISS), to inform countermeasure and mitigation plans for future deep-space missions. Thus far, only 12 astronauts from the Apollo missions have traveled beyond the Earth's protective geomagnetic Van Allen radiation belt, which shields the Earth from galactic cosmic rays (1–3). Notably, previous studies showed that Apollo astronauts exhibit higher morbidity/mortality rates associated with cardiovascular disease (CVD) compared to non-flight astronauts and astronauts who flew only LEO missions (4). Considering observed health risks may be modified by several spaceflight factors (i.e., duration, distance from Earth, etc.), further studies are needed to elucidate underlying molecular mechanisms driving cellular dysfunction that may lead to disease to inform mitigation and countermeasure plans.

Accumulating evidence shows that physical and psychological factors associated with spaceflight (isolation, confinement, acceleration at launch, sleep deprivation, microgravity, and space radiation) may dysregulate astronauts' genetic, transcriptional, and metabolic profiles (5). For example, transcriptional profiling of whole blood collected from six astronauts who flew relatively short (up to 14 days) Shuttle missions showed genes involved in DNA repair (*XRCC1, HHR23A*), oxidative stress (*GPX1*), and chaperones (*HSP27, HSP90AB1*) to be downregulated post-spaceflight and proto-oncogene *C-FOS* to be upregulated (6). In addition, our group has demonstrated that circulating levels of cell-free mitochondrial DNA (cf-mtDNA) are elevated after relatively short Shuttle missions and are associated with elevated transcripts of DNA damage, oxidative stress, and inflammatory markers in peripheral blood mononuclear cells (PBMCs) (7). Furthermore, NASA's Twin study found that year-long LEO spaceflight was associated with altered telomere dynamics inflight, differential expression of genes related to DNA repair, immune function, oxidative stress, apoptosis, and mitochondrial respiration in PBMCs, and altered genome-wide DNA methylation in CD4 and CD8 cells (8). Thus, such genomic, epigenomic, or transcriptional alterations may provide metrics for monitoring astronauts' health before, during, and after spaceflight.

Exosomes are small extracellular vesicles (sEVs) measuring 30–140 nm with a notable role in autocrine and paracrine signaling and thus mediate essential biological functions involved in cell physiology and human diseases (9–11). Their roles in intracellular communication can be attributed to their cargo that contains various biomolecules like RNA, DNA, proteins, and other metabolites (12, 13). Several studies have implicated exosomes in various diseases and processes, including (1) immune process (i.e., cytokine production, antigen presentation, and NK-cell activation) (14–18), (2) CVD (atherosclerosis, myocardial ischemia and reperfusion injury, heart failure, and cardiomyopathy) (19–24), and (3) cancer (22, 25, 26), etc.

Considering their role in disease pathogenesis, we hypothesize that exosomes may mediate the harmful effects of spaceflight-associated stressors by altering the gene expression profile of various cells throughout the body. In this study, we found that astronaut-derived exosomes increased inflammation and oxidative stress in the AC16 human cardiomyocyte cell line. Interestingly, we uncovered enrichment in gene sets associated with SUZ12, vitamin D receptor (VDR), and H3K27me3 in AC16 cells treated with exosomes isolated at three days post landing (ExoR+3) compared to those treated with exosomes isolated 10 days before the flight (ExoL-10). Further, analysis of the VDR promoter revealed that ExoR+3 represses VDR expression by promoting H3K27me3 enrichment in the VDR promoter in an EZH2-dependent manner. Taken together, our results suggest that VDR agonists may be used to reduce exosome-mediated oxidative stress and inflammation in astronauts.

## Materials and Methods

### Astronaut Samples

Blood was sampled 10 days before launch (L-10) and 3 days after return (R+3) from 3 astronauts (Sample ID: D12, D13, and D14) who flew short (up to 14 days) Shuttle missions between 1998 and 2001. Information regarding de-identified blood samples and the binned age of the crew members at launch are provided in Table 1. All samples were stored at −80°C until use.

**Table 1:** Information regarding de-identified blood samples from 3 astronauts, including the binned age of the crew members at launch and sample ID number.

**Sample ID #**	**Binned age at launch in years**
D12	≥50
D13	<40
D14	<40

### Thrombin Plasma Preparation for Exosome Precipitation

Exosomes were isolated from blood plasma samples of 3 astronauts (Sample ID: D12, D13, and D14) at L-10 and R+3 using the ExoQuick Plasma prep and exosome precipitation kit (Cat # EXOQ5TM, System Biosciences, CA, USA), as previously described (7). Briefly, thrombin was added to the plasma and incubated for 5 min at room temperature. Next, samples were centrifuged at 10,000 rpm for 5 min, and the supernatant was incubated with the exosome precipitation solution for 30 min at 4°C. Samples were centrifuged for 30 min at 1,500 × g at 4°C, and the pellet was dissolved in 100 μl of sterile 1 × PBS.

### Exosome Uptake

Isolated sEVs were labeled using an SBI's ExoGlow-Membrane™ EV Labeling kit (Cat # EXOGM600A-1, System Biosciences, CA, USA) according to the manufacturer's protocol.

### Cell Culture and Treatment

AC16 human cardiomyocyte cell line was cultured in DMEM/F12 containing 2 mM L-Glutamine, 10% exosomes-free FBS, and 1% Penicillin/Streptomycin Solution. Based on our exosome uptake data, *in vitro* assays were performed using ExoL-10 and ExoR+3 isolated (10^8^ particles/mL) from Astronaut D14. After 24 h (post-sEV treatment), cells were co-treated with either GSK126 (1 μM, SelleckChem) for an additional 48 h, a potent EZH2 inhibitor, or paricalcitol (10 nM, SelleckChem), a synthetic vitamin D analog, for an additional 24 h (Supplementary Figure 1).

### RNA Isolation

For Affymetrix microarrays, total RNA was extracted from AC16 cells treated with astronaut-derived ExoL-10 and ExoR+3 (10^8^ particles/mL) for 24 h using the RNeasy Mini Kit (Qiagen), according to the manufacturer's protocol. High-quality RNA was isolated using RNeasy Mini spin columns and eluted using molecular-grade water (RNAse/DNAse free).

### Affymetrix Microarrays and Data Analysis

All samples first underwent quality control assessment to ensure successful library preparation. RNA sample quality was assessed by NanoDrop and Agilent 2100 BioAnalyzer. Samples with RNA integrity number (RIN) above 7, OD260/280: 2, and OD260/230 ≥ 2 were used. In addition, RNA degradation and contamination were monitored on 1% agarose gels before Affymetrix microarrays. DEGs were analyzed using the integrative web-based gene list enrichment analysis tool ENRICHR (http://amp.pharm.mssm.edu/Enrichr/, accessed 09/2020), followed by transcription factor (TF) enrichment analysis using the Encyclopedia of DNA Elements (ENCODE)/ChIP-X Enrichment Analysis (CheA) database. In addition, the NIH Roadmap Epigenomics Consortium that integrated epigenomic maps were generated from ChIP-seq datasets obtained using 127 human tissues and primary cells with antibodies targeting over 30 different histone modification marks.

### Oxidative Stress

AC16 cells were treated either with ExoL-10 or ExoR+3 (10^8^ particles/mL) for 24 h and then stained with CellROX™ Green Reagent for oxidative stress detection at the following time intervals: without cotreatment (sEVs alone) at 24 h post-sEV treatment, co-treatment with GSK126 (72 h post-sEVs), co-treatment with Paricalcitol (48 h post-sEVs) (Supplemental Figure 1). CellROX^®^ Green Reagent is a novel fluorogenic probe for measuring oxidative stress in live cells with absorption/emission maxima of ~485/520 nm. Briefly, AC16 cells were incubated with CellROX^®^ Green Reagent at a final concentration of 5 μM for 30 min at 37°C. Then, the cells were washed three times with PBS and analyzed by traditional fluorescence microscopy and microplate-based fluorimetry.

### Western Blot

AC16 cells were collected and resuspended in RIPA lysis buffer (ThermoFisher) containing a Protease and Phosphatase Inhibitor Cocktail (Roche) and analyzed as previously described (27). Briefly, samples were centrifuged, and the supernatants were collected. Protein concentration was determined by BCA assay (Sigma-Aldrich). Fifty microgram total protein was loaded on a 4–20% mini-protean TGX precast protein gel and transferred to a polyvinylidene difluoride membrane. The membrane was blocked with 5% skim milk and probed with the primary antibodies diluted at 1/1,000 (EZH2, Cell signaling # #5246S; GAPDH, Invitrogen, #AM4300; H3K27me3, Cell signaling, #9733S, Histone H3, Cell Signaling, #9715S) overnight at 4°C followed by corresponding secondary HRP-conjugated antibodies (Cell signaling). The blots were developed using the SuperSignal West Dura Reagent (Thermo Scientific) and visualized using a BioRad imager.

### ChIP-qPCR

We performed ChIP-qPCR to measure the level of H3K27me3 in the VDR promoter in AC16 pre-treated for 24 h with ExoL-10 or ExoR+3 (10^8^ particles/mL) before co-treatment with GSK126 for 48 h (Supplementary Figure 1). At 72 h post-initial sEV treatment, AC16 cells were washed twice with cold PBS after removing the culture media, and chromatin was cross-linked immediately by adding 1% formaldehyde (Sigma-Aldrich, F8775) to the cell culture media and incubated for 10 min at room temperature. Next, cells were incubated with 1 × glycine for 10 min to quench excessive formaldehyde and rinsed twice with cold PBS. Cells were collected using a sterile cell scraper. Cells were resuspended and washed by centrifugation in sterile ice-cold PBS containing 1 × Halt protease inhibitor cocktail (ThermoFisher). After centrifugation, the cell pellet was resuspended using cell lysis buffer containing protease inhibitor cocktail, followed by the addition of Nuclear Lysis Buffer, and ChIP assay was carried out as recommended by the manufacturer using EZ-Magna ChIP™ Chromatin Immunoprecipitation Kit (Millipore, 17-10086). Samples were incubated with 5 ug of ChIP-grade antibody against H3K27me3 (Abcam, ab6002) overnight at 4°C using a rotating platform. Non-specific IgG was used as a control. After IP, the chromatin-antibody conjugates were washed sequentially with low salt, high salt, lithium chloride, and TE buffer. Then, samples were incubated with Proteinase K at 62°C for 2 h with shaking (1,400 RPM), followed by 95°C for 10 min. A ≈175 bp region within the VDR promoter was amplified using the following primers: forward—GGTAAACTTGGCTACTGAGGTC and reverse—GGAAACAAATACTTCTTGTTGCCC. Relative H3K27me3 enrichment in the VDR promoter sequence in input, ChIP, and IgG samples was quantified using the Fast SYBR Green qPCR system (Applied Biosystems). DNA isolated from negative IgG control IP samples showed undetermined to negligible DNA amplification for VDR promoter, ensuring antibody specificity. Enrichment of DNA sequence (VDR promoter) in IP samples was then calculated as a percentage of input (% input) within the sample. Fold change between groups was expressed as relative to control samples.

### Total RNA Isolation, cDNA Preparation, and Quantitative RT-PCR Analysis

For the mRNA expression analyses, total RNA was extracted using TRIzol™ (Invitrogen) and purified using RNeasy mini columns (Qiagen). According to the manufacturer's instructions, the cDNA synthesis kit (Applied Biosystems, Foster City, CA) was used to generate cDNA. Quantitative RT-PCR was performed using the PerfeCTa SYBR™ Green FastMix kit (Quantabio) according to the manufacturer's instructions. Samples were analyzed using the ABI Prism 7500 Real-Time PCR System (Applied Biosystems, Foster City, CA). Fold changes in gene expression were determined using the relative comparison method with normalization to GAPDH as an internal control. The primer sequences are provided Table 2:

**Table 2:** Primer sequences for RT-qPCR analysis.

**Application**	**Gene symbol**	**Species**	**Forward primer (5^′^-3^′^)**	**Reverse primer (5^′^-3^′^)**
RT-qPCR	EZH2	Human	TCCCGCTGAGGATGTGGATA	GGGCACGAACTGTCACAAGG
	GAPDH		CGACCACTTTGTCAAGCTCA	AGGGGAGATTCAGTGTGGTG
	IL1A		AAGAAGACAGTTCCTCCATTGATCA	CCTTGAAGGTAAGCTTGGATGTTTT
	IL1B		ATGATGGCTTATTACAGTGGCAATG	ATCTTCCTCAGCTTGTCCATGG
	IL6		ACAAGAGTAACATGTGTGAAAGCAG	ACTCTCAAATCTGTTCTGGAGGTAC
	IL8		TTGCCAAGGAGTGCTAAAGAACTTA	AGCTCTCTTCCATCAGAAAGCTTTA
	SUZ12		TGCATTGCCCTTGGTGTACT	GCAAATCCAGGTTGGCGATG
	TNF		CTTGTTCCTCAGCCTCTTCTCCTTC	GGGTTCGAGAAGATGATCTGACTG
	VDR		CTGCATCGTCTCCCCAGATC	GGTCGGCTAGCTTCTGGATC

### Statistical Analysis

Results are presented as mean ± standard error of the mean. Data were analyzed using an unpaired *t*-test for comparisons between means 1-way analysis of variance with the Tukey correction for comparisons between >2 groups. Statistical analysis was performed using GraphPad Prism software (GraphPad Software, Inc., La Jolla, CA). Differences were considered significant at *P* < 0.05. Corresponding symbols in the figures are ^*^for *P* < 0.05, ^**^for *P* < 0.01, ^***^for *P* < 0.001, and ns for not significant.

## Results

### Astronaut-Derived sEVs Promote Oxidative Stress and Inflammation Markers in AC16 Cells

**Figure 1:**
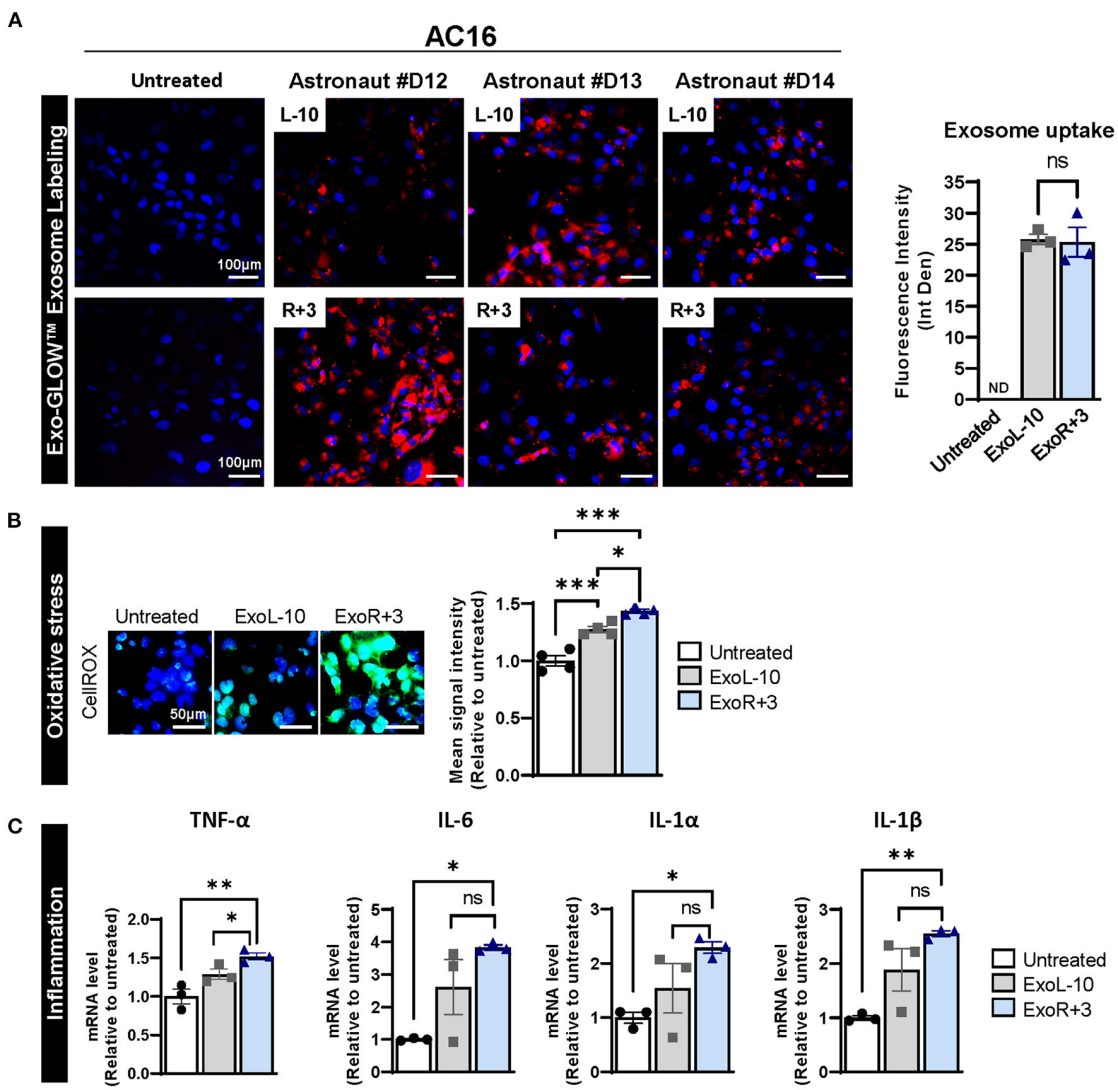
Astronauts-derived exosomes induce transcriptional changes in AC16. **(A)** Exosomes from L-10 and R+3 (ExoL-10, ExoR+3) were isolated from 3 astronauts (Sample ID: D12, D13, and D14) and labeled using an SBI's ExoGlow-Membrane™ EV Labeling kit. Next, labeled sEV were used to treat AC16 cells for 24 h. Representative pictures of AC16 cells are shown (left panel). Scale bars, 100 μm. Respective quantification is provided, right panel, *n* = 3. **(B)** Oxidative stress levels were measured using CellROX™ Green Reagent in untreated cells and AC16 cells treated for 24 h with ExoL-10 and ExoR+3 isolated from astronaut D14, *n* = 4. Scale bars, 50 μm. Cells were visualized by fluorescence microscopy and quantified using a fluorescent spectrophotometer. **(C)** Transcript levels of inflammation markers (TNF-α, IL-6, IL-1 α, and IL-1β) were measured by qPCR in the indicated conditions after 24 h, *n* = 4. Statistical significance was determined by one-way ANOVA followed by Tukey post-test. ^*^P < 0.05, ^**^P < 0.01, ^***^P < 0.001, ns, not significant.

To better understand the role of sEVs and their implication in spaceflight-associated adverse health effects, we isolated exosomes from the peripheral blood of 3 astronauts at L-10 (ExoL-10) and R+3 (ExoR+3). Exosomes were characterized by nanoparticle tracking analysis for size distribution and concentration and analyzed using an exosome-specific antibody array. Nanosight and Exo-Check Antibody Array data for the exosomes used in this study were recently published by Bisserier et al. (7). Next, AC16 cells were treated with L-10 and R+3 astronaut-derived exosomes that were labeled with Exo-Glow (ExoL-10, ExoR+3) for 24 h. Our results confirmed exosome uptake by AC16 cells (Figure 1A).

Considering the role of exosomes in various biological processes, we further assessed the effects of astronaut-derived exosomes on oxidative stress and inflammation. Our results show that both ExoL-10 and ExoR+3 increased oxidative stress 24 h post-treatment compared to untreated AC16 cells, though the degree of increase was slightly more pronounced in ExoR+3 treated cells compared to ExoL-10 treated cells (Figure 1B). We also analyzed the transcript levels of various inflammatory markers (TNF-α, IL-6, IL1-α, IL1-β), which revealed a significant increase in gene expression levels of all inflammatory markers in AC16 treated ExoR+3 for 24 h when compared to control untreated samples (Figure 1C).

### Enrichment Analysis of AC16 Treated With Astronaut-Derived Plasma Exosomes

**Figure 2:**
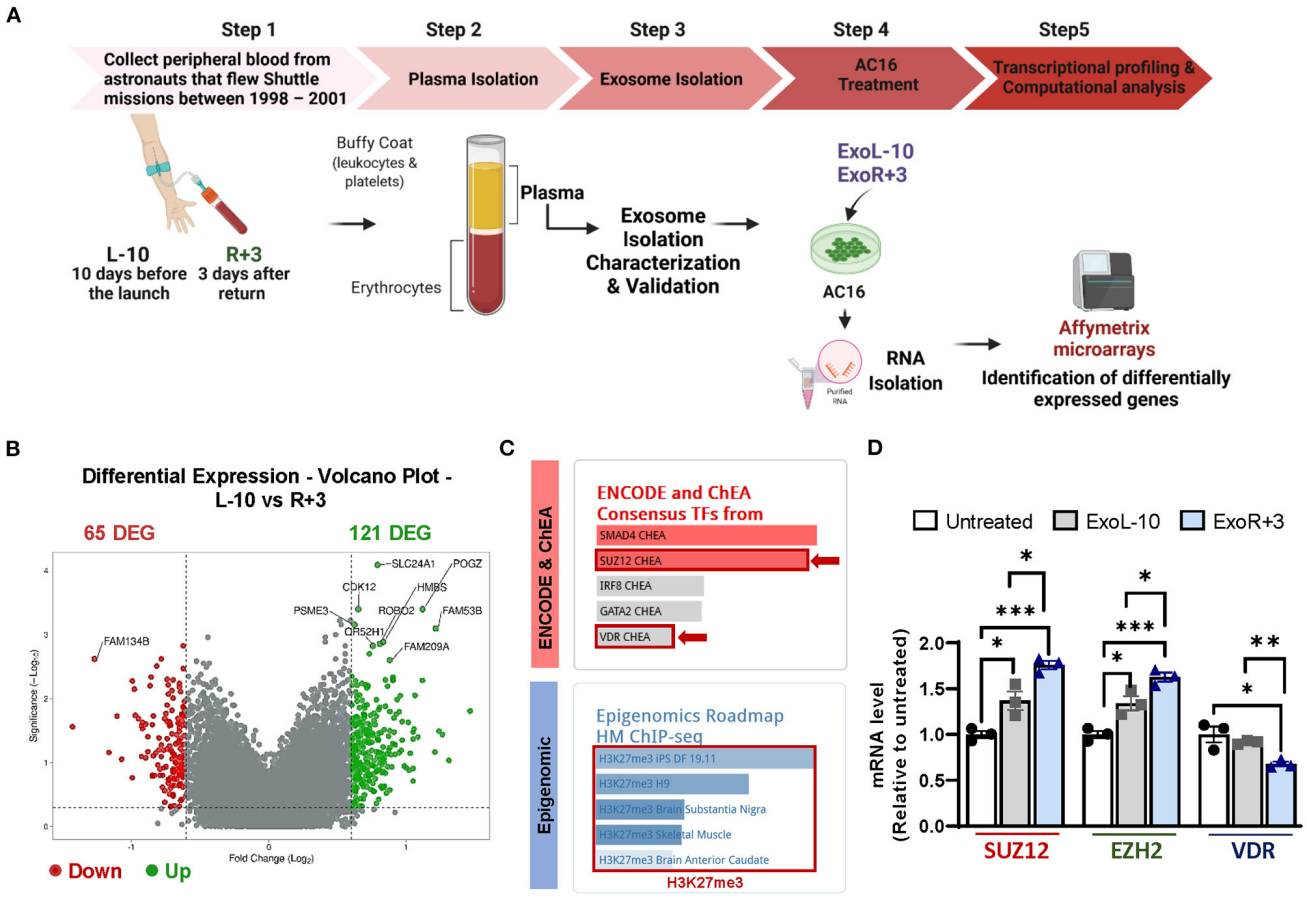
Identification of epigenetic and transcriptional changes in AC16 treated with astronauts-derived exosomes by Affymetrix microarrays. **(A)** Schematic representation of the experimental design. Blood was drawn 10 days before launch (L-10) and 3 days after return (R+3) from 3 different astronauts. The exosomes from L-10 and R+3 (ExoL-10, ExoR+3) were isolated from 3 different astronauts and then used to treat AC16 cells for 24 h. RNA from AC16 cells was isolated and analyzed using Affymetrix microarrays. Created with BioRender.com. **(B)** Volcano plots showing Log2-fold changes for the differentially expressed genes (DEG) and the statistical significance of each gene calculated after DEG analysis. Red points indicate significantly down-regulated genes; green points indicate up-regulated genes. **(C)** Gene list enrichment analysis was conducted using ENRICHR. Upper panel: transcription factor enrichment analysis (TFEA) using ENCODE/ChEA database. Lower panel: Epigenomic analysis using a gene-set library from the Roadmap Epigenomics Project. **(D)** qRT-PCR was performed on untreated cells and AC16 cells treated with ExoL-10- and ExoR+3 isolated from Astronaut D14 for 24 h, *n* = 3. Statistical significance was determined by one-way ANOVA followed by Tukey post-test. *P < 0.05, **P < 0.01, ***P < 0.001.

We treated AC16 cells with ExoL-10 and ExoR+3 isolated from 3 astronauts, and total RNA was isolated and analyzed for gene expression profiling using Affymetrix microarrays (Figure 2A). Computational analysis of microarray datasets revealed 186 differentially expressed genes (DEGs), 65 down- and 121 up-regulated, in the AC16 treated with ExoR+3 compared to AC16 treated with ExoL-10 (Figure 2B). Gene list enrichment analysis using ENRICHR and transcription factor (TF) enrichment analysis using ENCODE/ChEA database identified SMAD4, SUZ12, IRF8, GATA2, and VDR amongst the most enriched TFs (Figure 2C, upper panel). Polycomb repressive complex 2 (PRC2), formed by SUZ12, EZH2 (Enhancer Of Zeste 2 Polycomb Repressive Complex 2 Subunit), and EDD subunits, plays a key role in the coordination of Polycomb mediated gene silencing, chromatin compaction, and methylation of histone H3 at lysine 27 (H3K27me3) (28). SUZ12 plays an essential role in maintaining the integrity of PRC2 and the stability of EZH2 and thus maintains a critical role in modifying the histone methyltransferase activity of the complex. Specifically, SUZ12 is responsible for catalyzing H3K27me3.

Thus, we used a gene-set library associated with histone modifications extracted from the Roadmap Epigenomics Project (Figure 2C, lower panel) for our 186 DEGs. We found significant enrichment in the repressive histone methylation mark H3K27me3 (Figure 2C, lower panel). qRT-PCR validation confirmed that ExoR+3 astronaut-derived exosomes significantly increased SUZ12 and EZH2 expression while repressing VDR expression, compared to ExoL-10 treated AC16 (Figure 2D). Interestingly, SUZ12 and EZH2 were also upregulated in ExoL-10-treated AC16 compared to untreated cells.

### Enrichment of H3K27me3 in VDR Promoter and Its Functional Consequence

**Figure 3:**
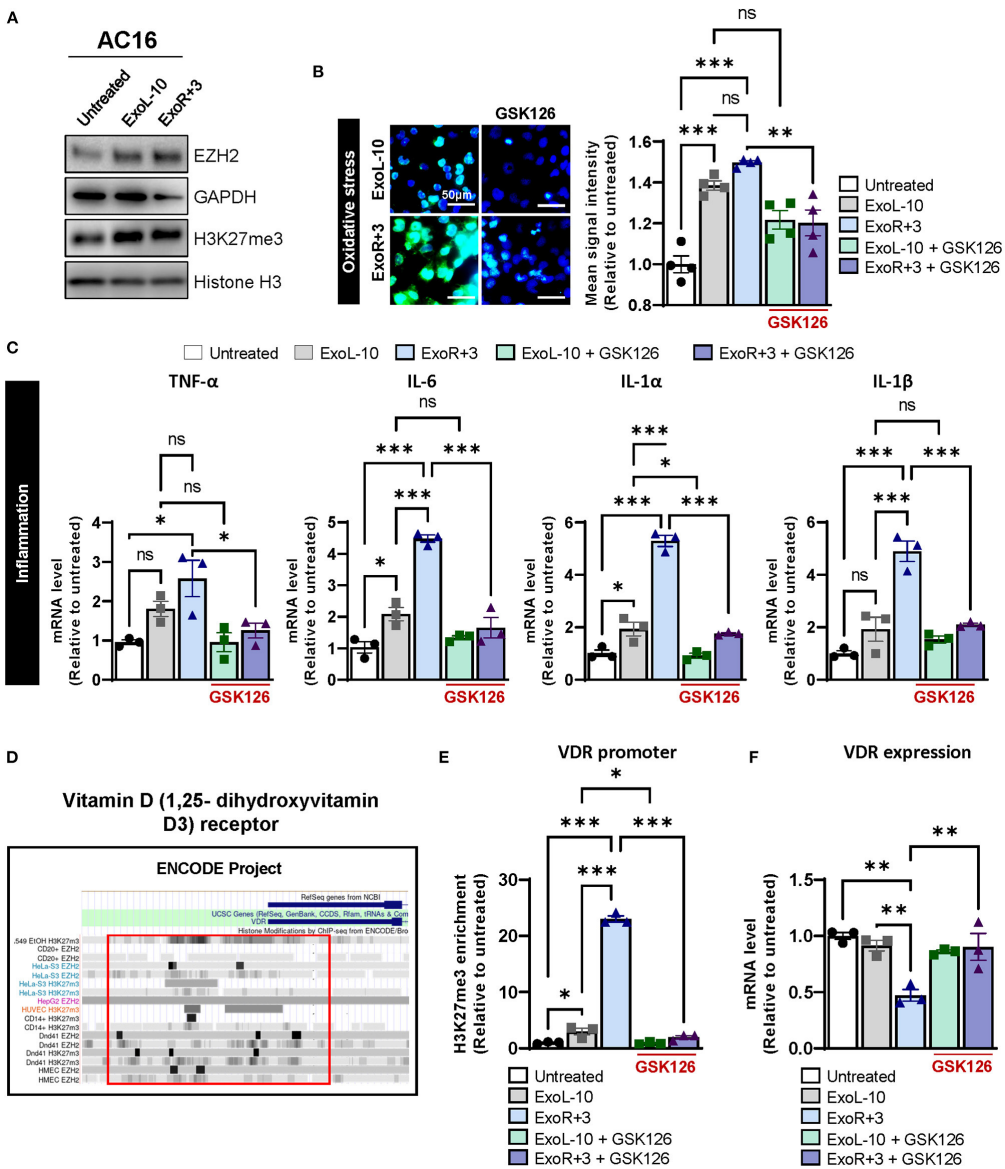
Epigenetic regulation of VDR *via* H3K27me3 in AC16 treated with astronauts-derived exosomes. **(A)** AC16 cells were treated with ExoL-10 or ExoR+3 (10^8^ particles/mL) for 24 h and analyzed by immunoblotting for EZH2, H3K27me3. Histone H3 and GAPDH were used as loading controls. A representative immunoblot is shown. **(B)** Oxidative stress levels were measured using CellROX™ Green Reagent in untreated cells and AC16 cells pretreated for 24 h with ExoL-10 and ExoR+3-isolated from astronaut D14 alone or combined with GSK126 (1 μM, treatment at 24 h post-sEVs) for an additional 48 h, *n* = 4. Cells were visualized by fluorescence microscopy and quantified using a fluorescent spectrophotometer. Scale bars, 50 μm. **(C)** Transcript levels of inflammation markers (TNF-α, IL-6, IL-1 α, and IL-1β) were measured by qPCR in AC16 pretreated for 24 h with ExoL-10 and ExoR+3 alone or cotreated with GSK126 (1 μM) for an additional 48 h, *n* = 3. **(D)** VDR promoter was analyzed using H327me3-ChIP ENCODE datasets. **(E)** H3K27me3 abundance in the VDR promoter was measured by ChIP-qPCR in AC16 cells pretreated for 24 h with ExoL-10 and ExoR+3 alone or in combination with a specific inhibitor against EZH2 (GSK126; 1 μM) after 48 h. **(F)** VDR mRNA levels were measured by qPCR in AC16 treated with ExoL-10 and ExoR+3 alone or combined with GSK126 (1 μM) 72 h after initial exosome treatment. Statistical significance was determined by one-way ANOVA followed by Tukey post-test. *P < 0.05, **P < 0.01, ***P < 0.001, ns, not significant.

To better define the contribution of PRC2 to the epigenetic regulation of VDR expression after spaceflight, we treated AC16 cells with ExoL-10 and ExoR+3 derived from astronaut D14 for 24 h and analyzed by immunoblotting for EZH2 and H3K27me3 levels. Our results demonstrated that both astronaut-derived ExoL-10 and ExoR+3 treatment potentiated EZH2 expression and increased global levels of H3K27me3 in AC16 cells (Figure 3A). Next, we used GSK126, a potent and highly selective EZH2 methyltransferase inhibitor, to examine the impact of pharmacologic inhibition of PRC2-EZH2 activity on oxidative stress and inflammation induced by astronaut-derived exosomes. Interestingly, we found that after 48 h of GK126 treatment, EZH2 inhibition alleviated oxidative stress and the expression of pro-inflammatory markers in cells treated in combination with ExoR+3 (Figures 3B,C). Finally, we also analyzed previously published H3K27me3–ChIP ENCODE datasets to understand the role of PRC2 on epigenetic regulation of VDR expression. We found enrichment in H3K27me3 within the VDR promoter (Figure 3D), suggesting that astronaut-derived exosomes may repress VDR expression by promoting H3K27me3 abundance within the promoter region. Furthermore, assessment of enrichment of H3K27me3 in the VDR by ChIP-qPCR showed that ExoR+3 significantly increased H3K27me3 enrichment within the VDR promoter region in AC16 compared to untreated cells or ExoL-10-treated cells (Figure 3E). Moreover, we used GSK126 to examine the impact of pharmacologic inhibition of PRC2-EZH2 activity on H3K27me3 abundance in the VDR promoter. Our results demonstrated that impairment of the PRC2-EZH2 activity significantly decreased EZH2-induced H3K27me3 enrichment in the VDR promoter (Figure 3E) and was associated with the restoration of VDR expression (Figure 3F). Collectively, our data suggested that astronaut-derived exosomes may repress VDR expression in an H3K27me3-dependent manner.

### Paricalcitol Inhibits the Expression of Oxidative Stress and Inflammation Markers in AC16 Treated With Astronaut-Derived Exosomes

**Figure 4:**
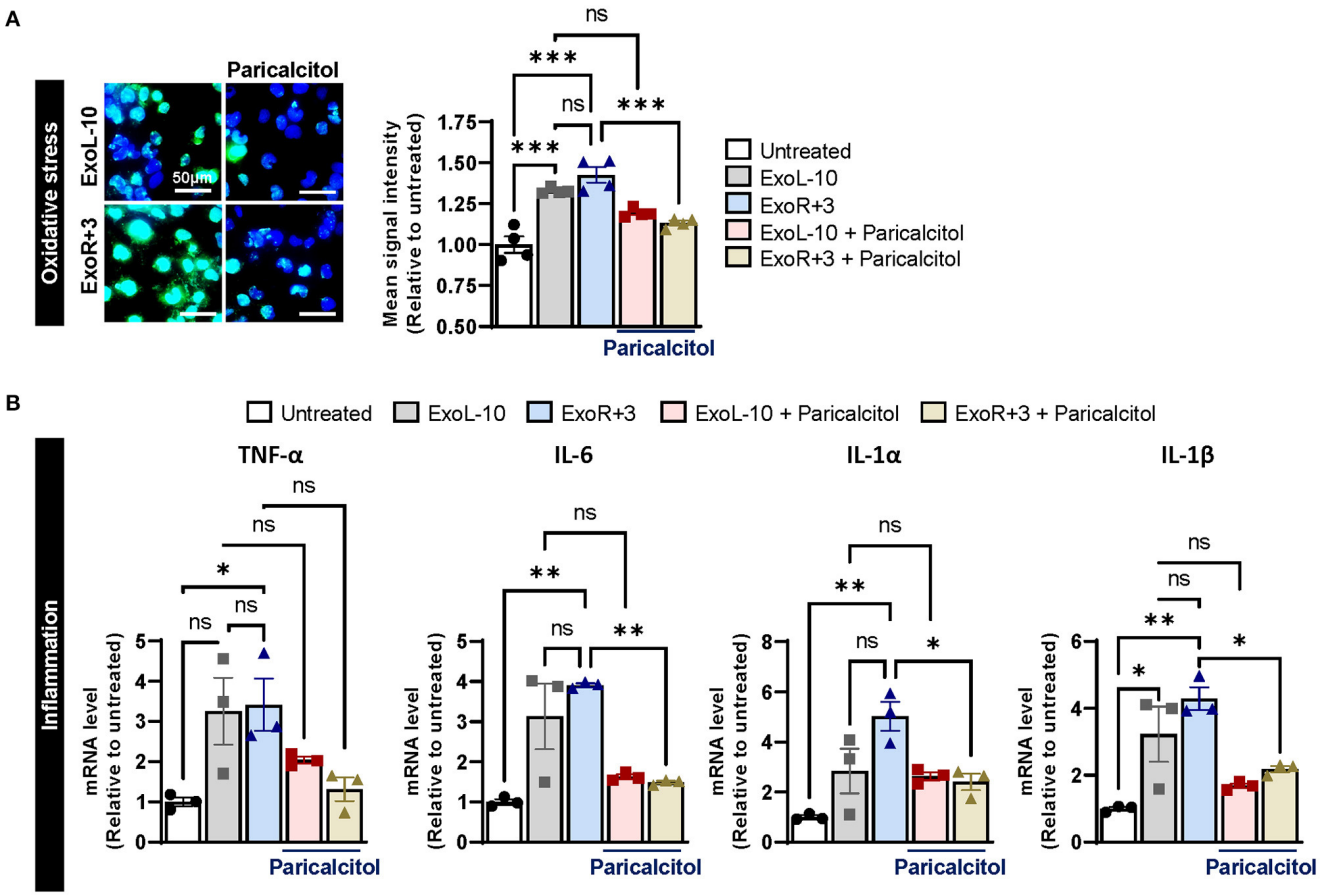
Supplementation with VDR agonist paricalcitol inhibits sEVs-induced oxidative stress and inflammation in AC16. **(A)** Oxidative stress levels were measured using CellROX™ Green Reagent in untreated cells and AC16 cells pretreated for 24 h with ExoL-10 or ExoR+3 from Astronaut D14 and paricalcitol (10 nM) after 24 h, *n* = 4. Cells were visualized by fluorescence microscopy and quantified using a fluorescent spectrophotometer. Scale bars, 50 μm. **(B)** Transcript levels of inflammation markers (TNF-α, IL-6, IL-1 α, and IL-1β) were measured by qPCR in AC16 pretreated for 24 h with ExoL-10 and ExoR+3 isolated from Astronaut D14 alone or with paricalcitol (10 nM) after 24 h, *n* = 3. Statistical significance was determined by one-way ANOVA followed by Tukey post-test. *P < 0.05, **P < 0.01, ***P < 0.001, ns, not significant.

Previous studies suggested that vitamin D deficiency or defective VDR may be detrimental to cardiovascular health and other diseases, such as cancer, infectious and autoimmune diseases. To evaluate the therapeutic potential of vitamin D supplementation in astronauts, we used a synthetic vitamin D analog paricalcitol to selectively activate vitamin D responsive pathways in AC16 cells pretreated with ExoL-10 and ExoR+3 for 24 h. Paricalcitol treatment significantly inhibited oxidative stress (Figure 4A), and inflammation (Figure 4B) at 48 h in AC16 treated with ExoR+3. Notably, our study suggests that vitamin D supplementation in astronauts may represent a strategic and efficient approach for mitigating health risks associated with space travel.

## Discussion

Spaceflight and its various stressors are associated with various adverse health effects for astronauts who flew short or long-duration LEO missions. These risks will be further modified during deep space exploration missions to the Moon and Mars, increasing the need to understand the mechanisms behind cell/tissue and an organ perturbation to inform future countermeasures and mitigation plans. Collectively, this pilot study suggests that relatively short LEO spaceflight may be associated with epigenetic silencing of VDR function, and supplementation with vitamin D may represent a strategic and efficient approach for mitigating space flight-associated health risks.

Considering PRC2's role in epigenetic regulation of downstream gene expression, its catalytic subunit EZH2 has emerged as a therapeutic target of interest. EZH2 overexpression and gain-of-function mutations are associated with aberrantly high H3K27 trimethylation levels and repression of target genes related to cell cycle progression, proliferation, apoptosis, autophagy, senescence, and inflammation (29–31). As a result, further studies have associated dysregulation of EZH2 with various diseases, including CVD (cardiac hypertrophy, fibrosis, atherosclerosis, ischemic heart disease, myocardial regeneration, cardiomyopathy, pulmonary arterial hypertension, atrial fibrillation) (26, 32–37), various solid cancers (lung, breast, endometrial, ovarian, nasopharyngeal, thyroid, liver, prostate, and glioblastoma) (8, 17, 38–40), as well as hematopoietic cancers (Non-Hodgkin's, large B-cell, and follicular lymphoma) (41, 42). Preclinical studies investigating inhibition of EZH2, primarily with inhibitors GSK126 and EPZ6437 (Tazemetostat), have been promising in both CVD and cancer (30, 39, 43–47) and have led to early phase clinical trials in the context of various lymphomas and advanced solid tumors (NCT01897571), mesothelioma (NCT02860286), sarcoma (NCT02601950). Importantly, our report emphasizes the importance of characterizing the exosomal protein and RNA cargo to understand better how astronaut-derived exosomes affect histone modifications, gene expression, and biological responses in recipient cells. Our group has previously purified exosomal RNA and analyzed the transcriptome changes associated with spaceflights by small RNA-sequencing (sRNAseq). Our analysis of the sRNASeq datasets suggested that miR-214 (FDR 0.05; Log2fold−2.3) and miR-128 (FDR 0.1; Log2 fold−1.5), whose expression negatively correlates with EZH2 and SUZ12, respectively, were significantly downregulated 3 days post-flight (R+3) compared to baseline (L-10) (Supplementary Table 1). Downregulation of these miRs may increase the expression of both PRC2 components, thereby promoting the activation of the PRC2 complex and H3K27me3 levels in AC16 cells treated with exosomes isolated from astronauts at R+3. Other known miRNA (miR-144, miR-625, miR-31, miR-200b) and lncRNA (Meg3, SNHG6) targets of EZH2 and SUZ12 were detected, though there was no significant change in expression of these non-coding RNA post-flight. Therefore, further studies validating the role of these miR in counteracting EZH2-mediated epigenetic regulation may be of interest. We also noted that ExoL-10 and ExoR+3 treatments induced a different degree of response with regards to oxidative stress and inflammation in a time-specific manner. Indeed, increasing evidence indicates that epigenetic regulation is a dynamic and complex molecular mechanism that can regulate chromatin structure and accessibility within a few minutes (48, 49). Indeed, histone modifications, such as methylation and/or acetylation, are a highly specific epigenetic mark that is controlled by different regulatory processes in a time-sensitive window with gene-specific transcriptional outcomes (50). Because epigenetic modifications determine which genes are upregulated on a transient timescale upon cellular activation but also control stable gene expression on a timescale that extends beyond the initial stimuli (51), time-specific differences in response to sEVs treatment may suggest that oxidative stress and inflammation may be epigenetically regulated in AC16 cells. In addition, we also observed that the longer the treatment course with sEVs, the stronger the induction of oxidative stress and inflammatory response. For example, we also noted that differences in response between ExoL-10 and ExoR+3 treatment are more pronounced at 72 h compared to 24 or 48 h. Further investigation would be needed to increase the sample size and draw more robust conclusions to fully evaluate the therapeutic potential of this pathway.

Our study showed that astronaut-derived exosomes may repress VDR expression by promoting EZH2-mediated H3K27me3 in AC16 cells. Growing evidence from several preclinical studies in rodents and observational clinical reports suggests that vitamin D deficiency or defective VDR is associated with adverse cardiovascular outcomes (52). Furthermore, low vitamin D responsiveness is described as a major risk factor for hypertension, myocardial infarction, heart failure, peripheral arterial disease, and strokes (53). VDR-null mice exhibit dysregulation of the renin-angiotensin system (RAS), resulting in high blood pressure, cardiac hypertrophy, and overall elevated heart weight-to-body weight ratio (54). Further studies aimed at elucidating the pathophysiologic mechanisms underlying the role of vitamin D hormone 1α,25-dihydroxy vitamin D3 (1,25(OH)2D3) showed VDR-deficient mice exhibited lower bioavailability of nitric oxide (NO) due to reduced endothelial NO synthase expression, resulting in increased endothelial dysfunction, arterial stiffness, aortic impedance and remodeling, and overall impaired systolic and diastolic heart function which was independent of the alterations in (RAS) (55). Chronic supplementation of 1,25(OH)2D3 has been shown to modulate vascular tone and lower blood pressure by reducing reactive oxygen species production and decreasing cyclooxygenase-1 (COX-1) mRNA and protein expression in spontaneously hypertensive rats (56). Several preclinical studies have aimed to address the translational implications of these findings. Two prospective cohort studies, one in 613 men and 1,198 women (healthcare professionals) without baseline hypertension and the other in 38,388 men and 77,532 women, suggested lower 1,25(OH)2D3 levels were associated with increased risk of hypertension independent of other covariates, including age, BMI, physical activity, race, and menopause status (57). Similarly, a meta-analysis conducted by Kunutsor et al. on 283,587 participants revealed that the top third of participants with the highest 1,25(OH)2D3 levels had a 30% reduced risk of developing hypertension (58). Although the association between 25(OD)D levels and risks of CVD is well-accepted, the therapeutic benefits of vitamin D supplementation remain largely unclear. Currently, there is an ongoing randomized clinical trial investigating the association between dietary vitamin D3 and omega-3-fatty acids (VITAL, NCT01169259) incidence of CVD or cancer. Principle results of VITAL show a significant reduction in cancer mortality in patients taking vitamin D supplements, but no effect was observed regarding an incidence of CVD endpoints (59). In line with these studies, recent evidence suggests that women with low vitamin D levels are at higher risk of breast cancer. Radiotherapy is commonly used in patients with breast cancer to reduce tumor size before surgery and tumor recurrence after surgery. However, recent studies suggest that radiotherapy also affects vitamin D metabolism and distribution in the body, which may ultimately counteract radiotherapy outcomes. Taken together, further studies are required to validate and elucidate the role of VDR deficiency in the context of CVD and cancer risks, especially when modified by covariates associated with spaceflight, such as radiation.

While we did observe the restoration of VDR expression following EZH2 inhibition with GSK126 as well as anti-inflammatory and anti-oxidant effects of paricalcitol supplementation in AC16 human cardiomyocyte cell line treated with astronaut-derived exosomes, further studies using a larger sample size and paired clinical data are warranted to validate these findings. Further longitudinal prospective studies using astronaut blood samples and paired clinical data are needed to validate whether circulating 1,25(OH)2D3 levels in astronauts are altered before, during, and after spaceflight and whether there would be a net benefit from vitamin D supplementation in reducing space flight associated health risks. Overall, our study provides a promising avenue to investigate vitamin D supplementation or EZH2 inhibitors as a method for mitigating spaceflight-associated chronic adverse health risks.

## Data Availability Statement

The datasets presented in this study can be found in online repositories. The names of the repository/repositories and accession number(s) can be found below: https://www.ncbi.nlm.nih.gov/geo/, GSE198322.

## Author Contributions

Conception and design of the research: MB, VG, PM, and DG. Acquisition of data: MB, AR, BL, VG, AS, AE, SA, PM, and DG. Analysis and interpretation of data: MB, NS, AS, AB, AE, MC, AA, and DG. Statistical analysis: AS and AE. Drafting manuscript: MB, NS, AB, VG, LH, and DG. Obtaining funding: MB, LH, and DG. All authors read and approved the final manuscript.

## Funding

This work was supported by the Translational Research Institute for Space Health Funds FIP0005 (to DG), National Aeronautics and Space Administration Grant 80NSSC19K1079 (to DG), American Heart Association Career Development Award 18CDA34110277, and startup funds from the Ohio State University Medical Center (to VG), American Heart Association-Post Doctoral Fellowship Grant 915681 (AR), National Institutes of Health Grant R01 HL133554 and American Heart Association 18IPA34170321 (to LH), NIH 5T32HL007824-22, and the Cardiovascular Medical Research and Education Fund (CMREF) (to LH and MB).

## Conflict of Interest

The authors declare that the research was conducted in the absence of any commercial or financial relationships that could be construed as a potential conflict of interest.

## Publisher's Note

All claims expressed in this article are solely those of the authors and do not necessarily represent those of their affiliated organizations, or those of the publisher, the editors and the reviewers. Any product that may be evaluated in this article, or claim that may be made by its manufacturer, is not guaranteed or endorsed by the publisher.

## Supplementary Material

Supplementary Figure 1 Schematic representation of the experimental design for assessment of oxidative stress, inflammation markers, VDR expression, and H3K27me3 levels in AC16 cells pretreated with ExoL-10 or ExoR+3 **(A)** alone, **(B)** co-treated with inhibitor GSK126, or **(C)** co-treated with Viatmin D analog Paricalcitol.

Supplementary Table 1 Transcriptome profile of astronauts-derived exosomes was analyzed by small RNA-sequencing (sRNAseq). Analysis of sRNAseq datasets (GSE193331) identified miR-214 (FDR 0.05; Log2fold −2.3), and miR-128 (FDR 0.1; Log2 fold −1.5) as potential regulators of the PRC2 activity 3 days post-flight (R+3) compared to baseline (L-10).
