# Supplementary figures and images for: Astronauts Plasma-Derived Exosomes Induced Aberrant EZH2-Mediated H3K27me3 Epigenetic Regulation of the Vitamin D Receptor
Source: Front Cardiovasc Med. 2022 Jun 16;9:855181. doi: 10.3389/fcvm.2022.855181 (PMC9243458; doi:10.3389/fcvm.2022.855181)

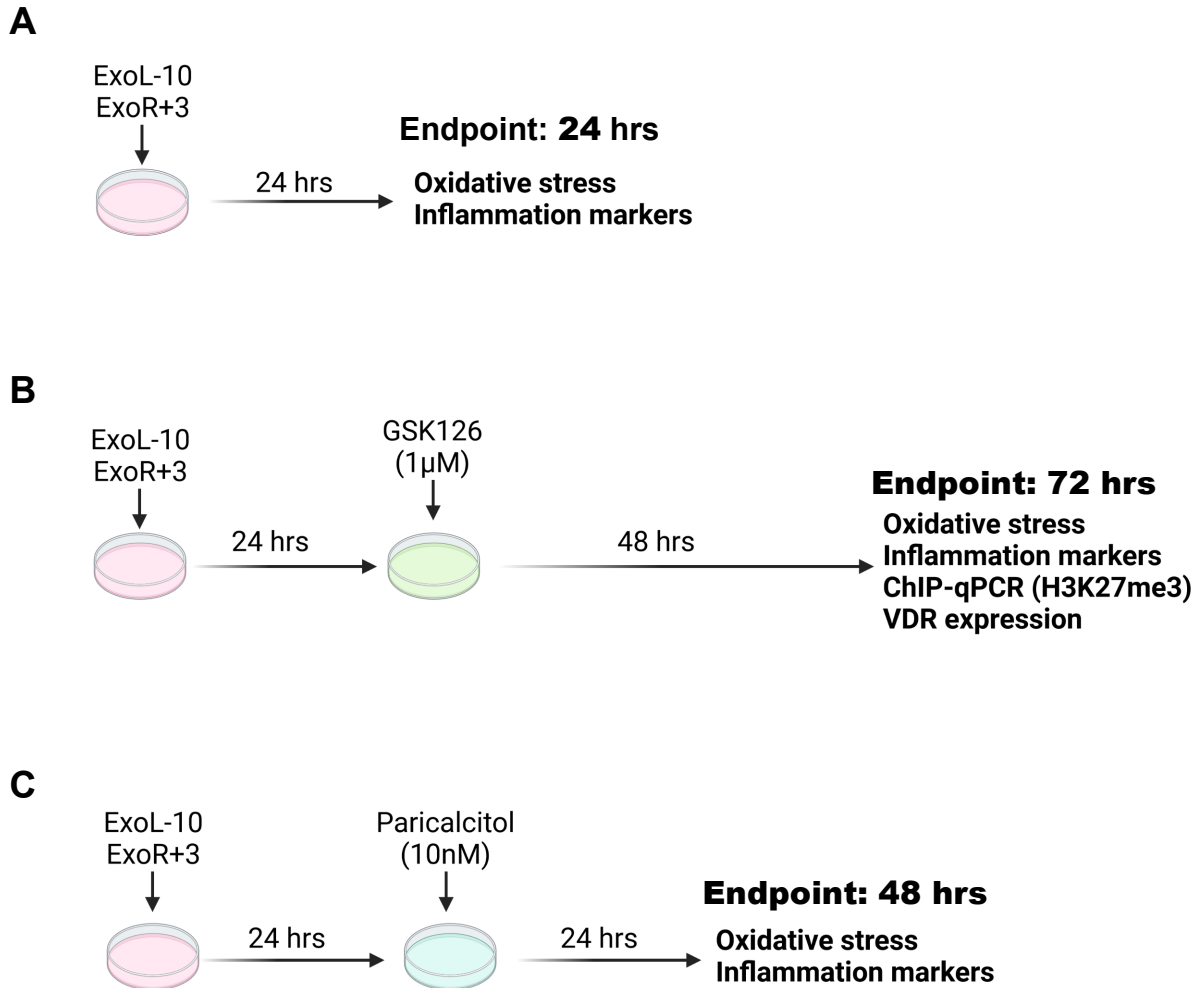

Supplement: Supplementary Figure 1 — Schematic representation of the experimental design for assessment of oxidative stress, inflammation markers, VDR expression, and H3K27me3 levels in AC16 cells pretreated with ExoL-10 or ExoR+3 (A) alone, (B) co-treated with inhibitor GSK126, or (C) co-treated with Viatmin D analog Paricalcitol. [file Image_1.pdf]
